# Radiomics and Back Pain

**DOI:** 10.1177/21925682251394282

**Published:** 2025-11-03

**Authors:** Jason Lin, Vinay Duddalwar, Michael M. Safaee

**Affiliations:** 1 Division of Biology and Biological Engineering, California Institute of Technology, Pasadena, CA, USA; 2 Radiomics Lab, Department of Radiology, University of Southern California, Los Angeles, CA, USA; 3 Department of Neurological Surgery, 5116 Keck School of Medicine of the University of Southern California, Los Angeles, CA, USA

**Keywords:** radiomics, back pain, review, diagnosis, prognosis

## Abstract

**Study Design:**

Narrative review.

**Objectives:**

Back pain is one of the leading causes of disability worldwide. While conventional imaging interpretation remains subjective and expertise-dependent, radiomics offers quantitative, data-driven analysis of medical images. We aimed to evaluate the current literature for the application of radiomics in: (1) soft tissue characterization, (2) hard tissue analysis, and (3) treatment outcome prediction in back pain conditions.

**Methods:**

We conducted a PRISMA-style literature search across PubMed, Google Scholar, Wiley, Springer, and IEEE Xplore, focusing on studies from the past 4 years. From 296 identified articles, 22 met inclusion criteria based on their use of radiomic methods and association with pain outcomes.

**Results:**

Current literature demonstrates that in many, but not all cases, using radiomics improves clinical models for soft and hard tissue diagnostics as well as for prognosis and treatment prediction. However, the improvements can be minor. There also exist limitations that prevent widespread clinical adoption of radiomics, including a lack of standardization in image acquisition/analysis protocols, homogeneity of patient populations studied, and inadequate integration with existing clinical imaging systems. Additionally, much current work is based on retrospective data instead of real-world data, where there is often an added complexity. Yet, there is increasing work in developing combined models where clinical features, demographics, and patient history are used to enhance the output and accuracy of radiomics.

**Conclusions:**

Radiomics can improve back pain diagnosis and treatment. Future directions should focus on developing generalizable radiomics models applicable to broad patient populations, imaging systems, and clinician-interpretable interfaces.

## Introduction

Back pain (BP) is one of the leading causes of disability worldwide and a prevalent global health problem.^1-4^ In 2020, low back pain affected an estimated 619 million people globally, with projections suggesting this number will rise to approximately 843 million by 2050.^5^ More concerningly, estimates suggest that up to 80% of the population will experience back pain at some point during their lifetime.^3,6^ While age-standardized rates have decreased modestly over the past 3 decades, the absolute number of affected individuals continues to rise due to population growth and aging.^1^ This underscores the urgent need for more effective diagnostic, preventative, and treatment strategies.

Current back pain diagnosis methods rely on patient history, physical examination, and imaging.^7^ While various imaging modalities can be used, each has specific clinical uses.^8^ However, image interpretation remains subjective and expertise-dependent, highlighting the need for more objective assessment methods.^9^

Radiomics is a quantitative approach to medical image analysis that enables the extraction of quantitative features not perceptible to the human eye.^10-14^ It has primarily been explored in oncology and is still most commonly applied to that field. In prostate cancer for example, it has been demonstrated that MRI-derived radiomic features can differentiate between clinically significant and indolent prostate cancer with higher accuracy than conventional radiological assessment alone.^15^ Recently, it has been shown that radiomics-based deep learning approaches can detect and segment osseous metastatic prostate cancer lesions on CT images.^16^ Yet, the potential for radiomics to act as an effective tool for clinical decision support in other clinical fields is becoming increasingly recognized.^11,17,18^ This approach takes advantage of the fundamental principle that medical images contain subtle elements that reflect the underlying pathophysiological processes, and that computer algorithms can be effective at finding these data.^19^

There are several key advantages to using radiomics in a clinical setting: first, it provides radiologists and treating physicians with more, better organized, and/or more accurate information to make diagnoses and create treatment plans. Second, when combined with machine-learning algorithms, radiomics can help physicians more accurately and efficiently sort through large quantities of medical images.^20^ Third, radiomics has been shown in selected instances to have better predictive abilities than traditional clinical markers, as higher-order radiomic features better correlate with pain mechanisms and functional outcomes than semantic measurements.^15,16^ Fourth, while traditional segmentation parameters such as volume, cross-sectional area, or percentage of intra-muscular fatty infiltration, have been shown to be useful in a clinical setting, radiomics analysis provides the physician with more information to make their diagnosis.^21^ This narrative review will explore the principles behind radiomic-based medical image analysis, explore the current literature regarding the application of radiomics to investigate back pain, and discuss the limitations and mitigation strategies involved in this approach.

## Radiomics Methodology

**Figure 1.**
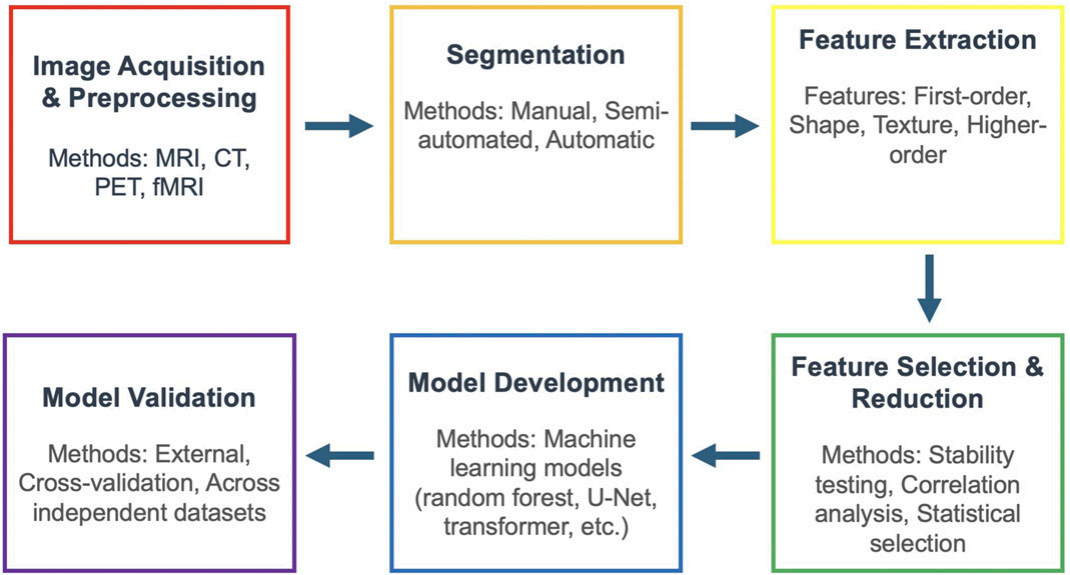
A Flowchart Demonstrating the Typical Workflow for Radiomics Analysis, Which Includes Image Acquisitions and Preprocessing, Segmentation, Feature Extraction, Feature Selection and Reduction, Model Development, and Model Validation

Since the first concept and workflow of radiomics was proposed by *Lambin et al* in 2012, the general framework of the technique has remained largely the same and can be broken down into the following categories: image acquisition and preprocessing, segmentation, feature extraction, feature selection and dimensionality reduction, and validation (Figure 1).^10,14,22^

### Image Acquisition

Medical image acquisition is the first step of the radiomics process. Modalities such as MRI, CT, positron emission tomography (PET), and conventional radiography can be used depending on the clinical context.^23^ The quality and consistency of acquired images significantly impact downstream data; consequently, standardization of acquisition parameters is essential, though impractical, for robust feature extraction.^24^ It has been shown that 80% of textural features extracted from PET and CT images have variations greater than 30% when the image grid size was changed.^25^ However, standardizing image acquisition parameters across a large number of institutions and medical conditions is difficult. A more feasible approach is to select reliable metrics that remain constant across vendors, acquisitions and possible temporal fluctuations. Recent works have demonstrated methods for identifying such stable radiomic features.^26-30^

### Segmentation

Segmentation involves the delineation of regions or volumes of interest (ROI/VOI). The objective is to isolate the anatomical or pathological structures from which radiomic features will be extracted. Segmentation methodologies can be defined along a continuum of manual, semi-automatic, and fully automatic approaches, with the latter being more suited for large datasets.^31^ However, there is no automatic segmentation algorithm that is suitable for all medical imaging types.^32,33^ It is generally agreed that the optimal way to perform segmentation while maintaining reproducibility is through a semi-automatic process.^31^ If semi-automated or manual segmentation is used, there must be clear documentation of inter-observer differences and quality checks to ensure accuracy and reproducibility.^34^

### Feature Extraction and Selection

There are over 1000 features that can be extracted using radiomics and they can be broadly divided into the following categories and as summarized in Table 1.^22,35^ Voxel intensity values represent the numerical measurements assigned to each three-dimensional pixel (voxel). First-order statistical features describe the distribution of voxel intensities within segmented regions without considering spatial relationships.^11^ These include intensity-based metrics (mean, median, minimum, maximum, standard deviation), histogram-based metrics (skewness, kurtosis, entropy, uniformity, energy), and percentile-based metrics.

**Table 1.** Summary of the Most Frequently Used and Analyzed Radiomic Features

Feature family	Description	Feature count (PyRadiomics v3.1.0)	Feature count (IBSI)^a^	Feature examples	Caveats
First-order/intensity statistics	Histogram‐based distribution of grey values, no spatial info	19	18	Mean, variance, entropy, 10th–90th percentiles	Strongly affected by bias-field correction and intensity normalization
Shape-based (3-D)	Geometry of the segmented object	16	29^b^	Volume, surface area, sphericity, elongation	Segmentation accuracy dominates reproducibility
Intensity-volume histogram (IVH)	Cumulative volume as a function of discretised intensity	Not listed	5	V10, V90, I10, I90, AUC	Requires re-binning
GLCM – Grey-level Co-occurrence matrix	Pair-wise occurrence of grey levels at given distance/angle	24	25	Contrast, correlation, inverse diff. norm., entropy	Sensitive to discretisation and resampling grid
GLRLM – Grey-level run length matrix	Length of consecutive voxels with same grey level	16	16	Short-run emphasis, long-run low GL emphasis	Unstable in very small ROIs
GLSZM – Grey-level size zone matrix	Size of 3-D zones of equal grey level, orientation-independent	16	16	Small-zone emphasis, zone percentage	Captures “mottled” texture; robust to rotation
GLDZM – Grey-level distance zone matrix	Zones of equal grey level and equal distance to ROI edge	Not listed	16	Large-distance emphasis, zone distance entropy	Adds boundary-centric heterogeneity
NGTDM – Neighbourhood grey-tone difference matrix	Difference between voxel and neighbourhood mean	5	5	Coarseness, busyness, complexity	Noise-sensitive without smoothing
GLDM – Grey-level dependence matrix	Size of local clusters of similar grey level	14	17	Dependence non-uniformity, low-dependence emphasis	Provides scale-adaptive heterogeneity
Higher-order filtered features	Any base family computed after LoG, wavelet, square, logetc.	Hundreds	Variable	Wavelet-HLH GLCM contrast, LoG-σ2 entropy	Strong feature selection needed

^a^Note that: IBSI counts are taken from the “Image features” chapter of the IBSI draft standard; they include some additional sub-families not implemented in PyRadiomics such as Local Intensity and extend GLCM to 25 descriptors.

^b^IBSI lists 29 morphology descriptors because it separates 2-D and 3-D variants; PyRadiomics v3 exports the 16 strictly 3-D metrics most commonly used.

Shape and morphological features characterize the two-dimensional and three-dimensional geometry of segmented regions independent of voxel intensity values. These features encompass volume measurements (total volume, surface area, surface-to-volume ratio), geometric descriptors (sphericity, compactness, elongation, flatness), and spatial characteristics (maximum diameter, major/minor axis lengths). Unlike intensity-based features, shape features demonstrate relative insensitivity to variations in acquisition parameters but exhibit significant dependence on accurate segmentation boundaries.^35^

Texture features characterize spatial patterns of voxel intensities: how intensities change, repeat, or correlate across neighboring voxels. Textural features are effective in capturing intratumoral heterogeneity but have also been shown to be sensitive to acquisition parameters and preprocessing steps.^25,36^

Higher-order and wavelet-based features expand the feature space by analyzing filtered versions of original images. Laplacian-of-Gaussian (LoG) filters smooth the image with a Gaussian kernel and then apply the Laplacian operator, sharpening edges and revealing subtle textural details at scale levels defined by the kernel width. Wavelet transforms take a complementary approach, recursively decomposing the image into paired high- and low-frequency sub-bands. The main advantage of wavelet transforms is that they support a systematic, multi-resolution analysis of patterns that may be overlooked at the native resolution.

The high dimensionality of radiomic features relative to typically limited sample sizes creates substantial risk of overfitting. Thus, reproducibility and stability are best achieved when features are ranked from a set of images acquired within a short period of time (eg, a few days apart) from the same patient cohort. Features that are highly correlated with one another can also be grouped together to reduce the number of features. However, a critical consideration in radiomics workflows is that not all pipelines measure the same metrics. This creates challenges in cross-platform validation and reproducibility. To address this issue, the Image Biomarker Standardization Initiative (IBSI) was established to address these standardization challenges by providing reference values for commonly used radiomic features and standardized computational workflows.^35^

Harmonization methods involve post-acquisition and post-processing techniques to reduce variability across different imaging systems and protocols. This is a critical technical approach for improving reproducibility in multicenter radiomics studies and can be broadly categorized into image domain and feature domain approaches.^37^ Image domain harmonization methods address variability at the pixel/voxel level before feature extraction and include standardization of acquisition protocols, post-processing of raw sensor-level data, data augmentation techniques using generative adversarial networks (GANs), and style transfer methods. However, while standardization of imaging protocols is common in clinical trials, this approach alone is insufficient for radiomics analysis as scanner-specific variations persist even with identical protocols. Feature domain harmonization is performed after radiomic feature extraction and primarily relies on statistical methods to adjust for batch effects. The most widely adopted approach is ComBat harmonization, a statistical method originally developed for genomics that uses empirical Bayes frameworks to estimate location and scale parameters.^38^ Enhanced variants, such as Nested ComBat, GMM ComBat, and M-ComBat, also exist and address limitations within the general ComBat procedure.^39^ However, it should be noted that harmonization may falsely negate true outlying biological signals and dampen clinically relevant variations. Further, the relationship between harmonization effectiveness and downstream predictive performance remains complex and context-dependent, with studies showing that improved harmonization performance does not guarantee improved predictive performance in downstream analyses.^39^

### Model Refinement and Validation

Radiomics model development typically follows either data-driven or hypothesis-driven approaches.^22^ Data-driven methods make no assumptions about individual features, treating all extracted features with equal weight during model construction. In contrast, hypothesis-driven approaches organize features into clusters based on predefined information content and clinical context.^19,22^

Proper validation is critical for establishing model reliability. External validation using independent datasets from different institutions is the gold standard.^40^ When external datasets are unavailable, internal validation techniques such as cross-validation are suitable, though less robust, alternatives.^41^

## Methods

We conducted a literature search of PubMed, Google Scholar, Wiley, Springer, and IEEE Xplore databases for studies that relate medical imaging features of the spine to back pain. The earliest and latest searches are April 20^th^ and June 2^nd^, 2025, respectively. The same search terms were used for all databased and included specific strings such as but not limited to “radiomics and back pain,” “radiomics and spine,” “radiomics and spine CT,” “radiomics and spine MRI,” “radiomics and spine treatment outcome,” “radiomics and vertebral CT,” “radiomics and vertebral MRI,” “radiomics and vertebral treatment outcome,” “radiomics and muscle fat infiltration,” and “radiomics and disc degeneration.” The complete list of keywords used during the search are detailed in Table 2. Reference lists of relevant papers were also screened. We included studies from 2021–2025, though we emphasize the increased importance and relevance of recent data.

**Table 2.** Details Regarding the PRISMA Style Search Plan

Databases	Keywords	Inclusion criteria	Exclusion criteria
1) PubMed	“Radiomics” AND “back pain” OR “soft tissue” OR “hard tissue” OR “muscle fat infiltration” OR “disc degeneration” OR “disc herniation” OR “vertebral fracture” OR “risk prediction”” OR “treatment outcome”	1) Studies analyzing quantitative features of lumbar or cervical spine tissues using radiomic methods	1) No pain outcome discussed
			2) Non-imaging studies
2) Google Scholar		2) Studies evaluating association between imaging features and presence/severity of back or neck pain	3) Animal studies
		3) Studies discussing clinical outcomes aimed at reducing spinal pain	4) Review articles
3) Wiley Online Library		4) Both cross-sectional diagnostic and prognostic studies (retrospective and prospective) are included	5) Non-English articles
4) Springer		5) English language publications	6) Conference abstracts without full publication
5) IEEE Xplore		6) Published from 2021-2025	7) Studies not specifically addressing spine-related conditions or pain assessment

**Figure 2.**
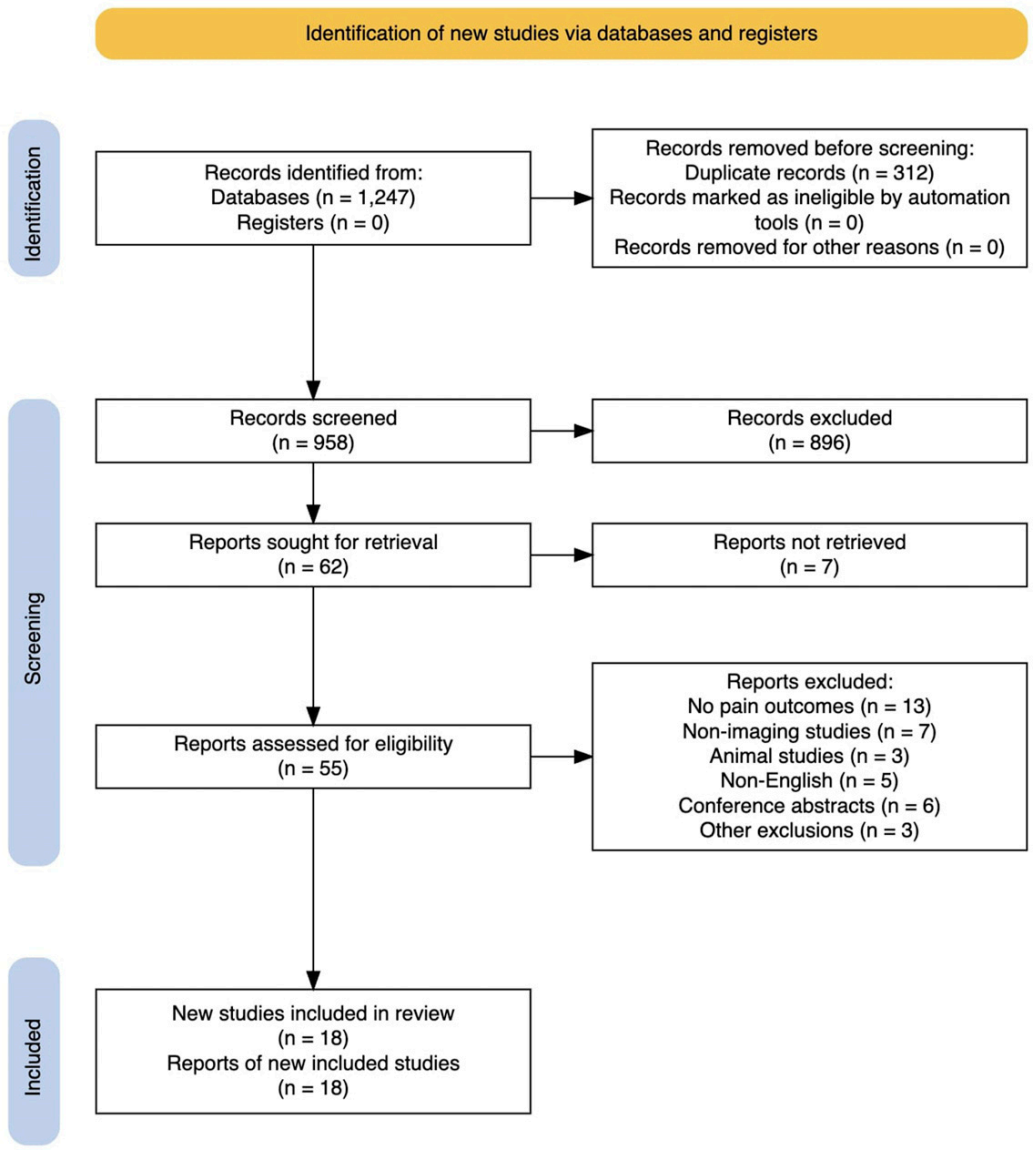
A Preferred Reporting Items for Systematic Reviews and Meta-Analyses (PRSIMA) Style Diagram Detailing Our Paper Selection Process. This Diagram was Created Using the R Package Tool Created by Haddaway et al.^42^

Studies were included if they: (1) analyzed quantitative features of lumbar or cervical spine tissues on imaging (MRI, CT, or other modalities) using radiomic methods; (2) evaluated an association between imaging features and the presence/severity of low back or neck pain; or (3) discussed a clinical outcome aimed at reducing spinal pain (eg, response to a specific treatment). Both cross-sectional diagnostic studies and prognostic or follow-up studies were eligible. We included both retrospective and prospective designs. Given our focus, most included papers applied radiomics or automated feature extraction, but we also considered notable studies using semi-quantitative measures of muscle or fat infiltration if they specifically linked imaging findings to pain. We excluded the following studies: (1) those that did not report any pain-related outcome or analysis, (2) non-imaging studies, (3) animal studies, (4) non-English articles, and (5) conference abstracts without full publication due to limited data. These steps are summarized in Table 2 and quantitatively summarized in Figure 2.

For each included study, we extracted information on author, year, patient population, imaging modality and sequences used, the target variable or clinical question answered (diagnosis of pain, prediction of outcome, etc.), the analytical approach (radiomics features, machine learning classifier or statistical model), and the main results (Tables 3–5). While we did not employ formal systematic quality assessment tools, we critically evaluated included studies for methodological rigor, clinical relevance, and potential bias. We acknowledge that this narrative approach, while providing comprehensive expert synthesis, may be subject to selection bias and does not provide the same level of evidence as systematic reviews or meta-analyses.

**Table 3.** Summarization of the Discussed Articles Regarding Soft-Tissue Characterization in Table Form

Author	Year	Title	Sample size & demographic	Imaging modality	Spinal region	Clinical purpose	Radiomic features selected	Best performing model	IBSI compliance	Harmonization	Performance metrics
Muhaimil et al.	2024	Role of artificial intelligence model in prediction of low back pain using T2 weighted MRI of lumbar spine	200 (100 with chronic LBP, 100 controls)	MRI (T2 weighted)	Lumbar vertebrae and intervertebral discs (L2–S1)	Predict LBP	20	Random forest, AdaBoost, deep CNN	NR	NR	0.83-0.88 AUC across lumbar levels (max 0.92 at L5–S1 disc). Deep CNN models yielded ∼84%-85% accuracy
Song et al.	2024	Prediction of low back fasciitis by machine learning method based on radiomics	380 with LBP (193 patients with superficial fasciitis and 187 normal)	MRI (T1 and T2 weighted)	L1-L5	Model to predict lumbar fasciitis	13	Combined clinical features and radiomics nomogram	NR	NR	0.97 AUC training, 0.96 AUC test
Yasin et al.	2024	MRI-based interpretable radiomics nomogram for discrimination between Brucella spondylitis and Pyogenic spondylitis	133 (68 with PS, 65 with BS)	MRI (FS-T2WI)	Not specified	Detect and differentiate PS and BS	9	Combined radiomics-clinical nomogram	NR	NR	0.962 AUC training, 0.859 AUC testing
Song et al.	2023	MR imaging radiomics analysis based on lumbar soft tissue to evaluate lumbar fascia changes in patients with low back pain	197 with chronic LBP	MRI (T1 and T2)	Thoracolumbar fascia	Diagnose fascia-related LBP (detect subtle fascial alterations)	7	Multivariate logistic regression (nomogram)	NR	NR	Differentiated LBP patients with fascial changes vs normals with AUC 0.92 (train) and 0.84 (validation)
Yasin et al.	2023	The potential of a CT-based machine learning radiomics analysis to differentiate Brucella and Pyogenic spondylitis	138 patients diagnosed with either PS or BS	CT (saggital)	L4-S1	Detect and differentiate PS and BS	15	KNN	NR	NR	0.91 AUC training, 0.87 AUC testing
Dieckmeyer	2021	Texture features of Proton density fat fraction maps from chemical shift encoding-based MRI predict paraspinal muscle strength	26	MRI (T2)	L2-L5	Diagnose and monitor diseases related to paraspinal musculature	22	Multivariate linear regression	NR	NR	Extension strength (R^2^ = 0.42; *P* < 0.001), flexion strength (R^2^ = 0.59; *P* = 0.001)

Note the following abbreviations: BP = back pain; LBP = low back pain; CBP = chronic back pain; CNN = convolutional neural network; FS-T2WI = sagittal fat-suppressed T2-weighted imaging; PS = Pyogenic spondylitis; BS = Brucella spondylitis; N/A = not applicable; NR = not reported.

**Table 4.** Summarization of the Discussed Articles Regarding Hard Tissue Characterization (Discs, Vertebrae, Endplates) in Table Form

Author	Year	Title	Sample size & demographic	Imaging modality	Spinal region	Clinical purpose	Radiomic features selected	Best performing model	IBSI compliance	Harmonization	Performance metrics
Hou et al.	2025	Clinical-radiomics nomogram construction from magnetic resonance imaging to diagnose osteoporosis: a Preliminary study	68 patients (33 with osteoporosis,35 with normal BMD)	X-ray, MRI (T1, T2)	Not specified	Predict osteoporosis	5	Clinical-radiomics nomogram	NR	NR	0.894 AUC, <0.0001 *P*-value. 91.43% sensitivity, 81.82% specificity
Wang et al.	2025	Integrating manual annotation with deep transfer learning and radiomics for vertebral fracture analysis	218 with VCF	CT, MRI (T2, STIR, TIRM)	Not specified	Diagnose acute and chronic VCF	28	Feature fusion model	NR	NR	AUC: 1.000 training, 0.964 test
Chen et al.	2024	Application of radiomics model based on lumbar computed tomography in diagnosis of elderly osteoporosis	182 elderly (132 normal, 50 with osteoporosis)	CT	L1-L4	Identify osteoporosis	14	KNN	NR	NR	AUC: 0.828 training, 0.796 test
Xie et al.	2024	MRI radiomics-based decision support tool for a personalized classification of cervical disc degeneration: a two-center study	435	MRI (T1 and T2)	Cervical intervertebral discs (C2–C7)	Grade cervical disc degeneration	924 total, top 10 selected	Random forest	“Most features…follow IBSI.”	Preprocessing referenced but not detailed; effect on performance NR	89.5% accuracy, 87.0% precision, 98.8% recall, 0.95 AUC match with radiologist grading
Waldenberg et al.	2023	Associations between vertebral localized contrast changes and adjacent annular fissures in patients with low back pain: A radiomics approach	61 with LBP	MRI (T1W, T2W, discography), CT	Vertebral bodies adjacent to discs (L1–S1)	Link vertebral marrow changes with painful annular fissures in discs	186 total, top 5 selected	Binary logistic regression using backward elimination	No	NR	*P* ≤ 0.002
Biamonte et al.	2022	Artificial intelligence-based radiomics on computed tomography of lumbar spine in subjects with fragility vertebral fractures	240 (58 with VF)	CT and XR-ray	Lumbar vertebral trabecular bone (D4-L4)	Detect skeletal fragility (osteoporotic VF risk)	93 total, top 20 selected	Linear support vector machine	Yes	NR	71.7% accuracy, 78% sensitivity, 69.6% accuracy, 0.8 AUC
Qiu et al.	2022	The value of radiomics to predict abnormal bone mass in type 2 diabetes mellitus patients based on CT imaging for paravertebral muscles	149 with type 2 diabetes mellitus	CT-QCT	Cancellous bone of T12 and L1 vertebral bodies	Predict low bone mass (osteopenia) in diabetics (risk stratification)	Muscle density and texture features on CT	Logistic regression model	NR	NR	AUC: 0.94 training, 0.90 test
											Accuracy: 87.8% training, 92.9% test
											Sensitivity: 83.3% training, 83.0% test
											Specificity: 93.0% training, 81.4% test
											PPV: 85.7% training, 77.4% test
											NPV: 77.4% training, 85.7% test

Note the following abbreviations: BMD = bone mineral density; VCF = vertebral compression fracture; STIR = short tau inversion recovery; TIRM = Turbo inversion recovery magnitude; BP = back pain; LBP = low back pain; CNN = convolutional neural network, PPV = positive predictive value; NPV = negative predictive value; NR = not reported.

**Table 5.** Summarization of the Articles Discussed Regarding Prognostic and Treatment Response Modeling in Table Form

Author	Year	Title	Sample size & demographic	Imaging modality	Target	Clinical purpose	Radiomic features	Algorithm (best performing)	IBSI compliance	Harmonization	Performance metrics
Fan et al.	2024	Machine learning assisting the prediction of clinical outcomes following nucleoplasty for lumbar degenerative disc disease	181 with LDDD	MRI (sagittal & axial T2)	Surgical outcome need (whether patient has risk factors necessitating surgery)	Identify surgical candidates among young LDH patients (who would benefit from early surgery)	Radiomics features (Rad_SAG, Rad_AXI) + deep CNN features (DL_SAG, DL_AXI) fused	Random forest	NR	NR	0.76 accuracy, 0.69 sensitivity, 0.83 specificity, 0.77 AUC
Lin et al.	2024	Radiomics based on MRI to predict recurrent L4-5 disc herniation after percutaneous endoscopic lumbar discectomy	128	MRI (T2W)	Recurrent disc herniation within 2 years post-PELD	Predict likelihood of recurrent LDH after minimally invasive surgery	1409 total, 18 selected	SVM, RF, and XG Boost	NR	NR	0.551-0.859 AUC, accuracy was 0.674-0.791, 0.647-0.729, and 0.674-0.718 for SVM, RF, and XG Boost respectively
Saravi et al.	2023	Clinical and radiomics feature-based outcome analysis in lumbar disc herniation surgery	172, previously underwent microdiscectomy for LDH	MRI (T2W)	1-year post-op outcome (pain relief vs persistent pain)	Predict surgical outcome (pain relief) after lumbar disc herniation surgery	Not provided	Various ML models	NR	NR	Best model (RBF-NN) improved to AUC 0.992 with radiomics (vs 0.970 with clinical data alone)
Chen et al.	2023	MRI feature-based radiomics models to predict treatment outcome after stereotactic body radiotherapy for spinal metastases	194 with spinal metastases treated with SBRT	MRI (T1, T2, fat-sat)	Tumor response after SBRT (RECIST progression vs non-progressive)	Predict tumor control and pain relief after spine SBRT	2264 texture features	Logistic regression model	“Most features…follow IBSI.”	Preprocessing referenced but not detailed; effect on performance NR	Pre-treatment MRI radiomics predicted treatment response with AUC ∼0.745-0.825, depending on sequence. Combined clinical+radiomics model had the highest AUC (0.828)
Llorián-Salvador et al.	2023	The importance of planning CT-based imaging features for machine learning-based prediction of pain response	135 receiving palliative radiotherapy for painful spine metastases	CT (radiation planning scans)	Pain palliation at 3 months post-RT (responder vs non-responder)	Predict which patients will achieve significant pain relief after radiotherapy	105 total, 4 selected	Support vector machine, random forest classifier, logistic regression	No	NR	Clinical features predicted with 0.80 AUC, combined model predicted with 0.75 AUC
Climent-Peris et al.	2023	Predictive value of texture analysis on lumbar MRI in patients with chronic low back pain	94	MRI (T2W)	Classifying patients whose BP did not improve by 30% or more	Predict which chronic LBP patients will fail to improve with standardized rehab	107 total, 45 selected	Random forest	Yes	NR	86% sensitivity, 57% specificity, 0.71 AUC. However, it poorly predicted disability outcomes (AUC ∼0.52)
Gui et al.	2022	Radiomic modeling to predict risk of vertebral compression fracture after stereotactic body radiation therapy for spinal metastases	74 patients treated with SBRT	CT + MRI (T1W, T2W)	Predict 1-year post-SBRT VCF	Predict fracture risk after spine stereotactic radiotherapy	93 total, 8 selected	Random forest	NR	NR	84.4% sensitivity, 80% specificity, 0.878 AUC.
Yu et al.	2022	Application of a nomogram to radiomics labels in the treatment prediction scheme for lumbar disc herniation	200 patients with lumbar disc herniation (pre-op)	MRI (T2W)	Predict need for surgery	Predict which LDH patients require surgical intervention	1083 features extracted, 11 selected	Multivariate logistic regression	NR	Image-domain: resampling to 3 × 3 × 3 mm, intensity normalization	91% accuracy, 91% specificity, 89% sensitivity, 0.93 AUC
Wakabayashi et al.	2021	A predictive model for pain response following radiotherapy for treatment of spinal metastases	69	Non-contrast enhanced CT	Pain response after radiotherapy	Predict significant pain relief from palliative radiotherapy	321 total, 44 selected	Random forest	NR	NR	82.6% accuracy, 76.20% specificity, 85.40% sensitivity, 0.848 AUC

Note the following abbreviations: BP = back pain; LBP = low back pain; VF = vertebral fracture; LDH = lumbar disk herniation; rLDH = recurrence of lumbar disk herniation; PELD = percutaneous endoscopic lumbar discectomy; LDDD = lumbar degenerative disc disease; NR = not reported.

Because many included studies used imaging-AI, we adapted elements from Transparent Reporting of a multivariable prediction model for Individual Prognosis Or Diagnosis (TRIPOD), Checklist for Artificial Intelligence in Medical Imaging (CLAIM), and quality assessment of diagnostic accuracy studies (QUADAS-2) to assess each study. For each included, we evaluated: (1) ground truth definition distinguishing between patient-reported pain outcomes (eg, numeric rating scale scores), imaging surrogates (eg, Pfirrmann grades), and intermediate findings (eg, vertebral fracture); (2) sample size, (3) feature selection methods (eg, LASSO); (4) machine learning algorithms employed; (5) validation strategies (eg, training/testing splits); (6) IBSI compliance status and software platforms; (7) harmonization procedures when reported; and (8) outcome classification.

## Literature Analysis

Accurate diagnosis of the anatomical or physiological source of back pain is often elusive. Below, we review studies that have used radiomics to detect or predict specific pathologies associated with back pain, broadly grouping them into those examining soft tissues (muscles, fascia) vs hard tissues (discs, bones), and then those aiming to predict outcomes or treatment responses.

Many studies quantify outcomes with Area Under the Curve (AUC) values: a threshold-independent metric that quantifies a model’s overall discriminative ability (eg, pain vs no pain). AUC values range from 0.5 (no discriminative ability) to 1.0 (perfect discrimination), with values above 0.7 generally considered acceptable, above 0.8 good, and above 0.9 excellent. AUCs from the included studies ranged from 0.52 to 0.99.^42^ Notably, the 8 studies predicting patient-important outcomes—those that directly matter to patients’ daily lives and wellbeing, such as pain relief, functional improvement, or return to work—showed more variable performance (AUC 0.52-0.99) compared to the 9 studies predicting imaging surrogates (AUC 0.76-0.96). 5 studies focused on treatment decisions or risk stratification.

### Soft Tissue Characterization

Radiomics has been applied to analyze the thoracolumbar fascia. Song et al developed an MRI-based radiomics model to detect lumbar fascial alterations in patients with chronic low back pain.^43^ Sagittal T2-weighted lumbar MRIs were used to extract texture features from the thoracolumbar fascia, yielding 7 radiomic features that distinguished patients with low back fasciitis from normal controls with an Area Under the Curve (AUC) of 0.92 in the training cohort and 0.84 in the validation cohort. This substantially outperformed the performance of models based on clinical features (eg, age, sex, sagittal Cobb angle) alone and provided a quantitative tool for the diagnosis of fascia-related pain. Similarly, Song et al developed a combined clinical-radiomics nomogram model to identify and predict low back fasciitis through soft tissue magnetic resonance.^44^

Another soft tissue focus is the paraspinal musculature. A study by Muhaimil et al used lumbar spine MRI radiomics to directly predict the presence of low back pain.^45^ They included 200 subjects and extracted features from T2-weighted MRI of lumbar vertebrae and intervertebral discs. Random Forest and AdaBoost classifiers achieved the highest performance, with AUC values between 0.83 and 0.88 across all lumbar vertebrae and intervertebral discs. Notably, this outperformed deep learning convolutional neural network (CNN) models (GoogleNet, ResNet18), which only achieved ∼84%-85% accuracy. A study by Dieckmeyer et al looked at texture features associated with paraspinal muscle strength.^46^ They found that kurtosis of the erector spinae and body mass index (BMI) are strong predictors of extension and flexion strength with *P* ≤ 0.001.

Spinal infections can also cause back pain. Yasin et al developed a model to differentiate between Pyogenic spondylitis (PS) and Brucella spondylitis (BS) with an AUC of 0.88 at an early stage from CT images.^47^ They also showed that this process can also be done via MRI images.^48^

Giaccone et al developed a fully automated pipeline for paraspinal muscle segmentation and fatty infiltration quantification, which could be used to process data prior to radiomics analysis.^49^ Though no study has yet applied radiomics to investigate steatotic fatty infiltration and back pain, it has already been shown that there are associations between intramuscular fat (IMF) and cross-sectional area (CSA) across the paraspinal muscles and self-reported back pain.^50-52^ Wesselink et al found that these differences were distributed broadly along the lumbar spine rather than being localized muscular changes, which challenges earlier notions that degeneration in the multifidus is only segmental.^50^ Thus, the above studies support fatty infiltration of spinal stabilizer muscles as an imaging biomarker of chronic low back pain. It has already been shown that radiomics markers can analyze fatty tissue and fatty infiltration into muscle fibers, though no radiomic study has yet looked at back pain from the perspective of fatty infiltration or subcutaneous fat composition.^53^ It remains to be determined whether reducing such fat (through exercise or other means) improves pain, but at a minimum, demonstrates the potential of paraspinal radiomics features to identify at-risk patients (Table 3).

### Hard Tissue Characterization

Radiomics has been applied to analyze intervertebral disc degeneration and herniation, which have been shown to cause low back pain.^54^ Xie et al created an MRI radiomics-based decision support tool for grading cervical disc degeneration.^55^ They extracted 924 texture and shape features from each segmented cervical disc on both T1-and T2-weighted MRI, and used feature selection (mRMR) plus machine learning to classify discs as mild vs advanced degeneration (per Pfirrmann grades). Results showed that higher-order textural heterogeneity features were the most predictive of degeneration severity. Further, the radiomics model based on T2-weighted images outperformed the T1-based model for disc grading. Waldenberg et al analyzed 61 chronic low back pain patients who underwent conventional MRI followed by CT-discography.^56^ After vertebral-body segmentation, 174 histogram- and texture-based radiomic features were extracted; a multilayer-perceptron model reduced this to 3 key vertebral marrow texture descriptors that distinguished levels neighboring painful annular fissures (discography-proven) from intact discs. The model achieved 83% accuracy, 97% sensitivity, 28% specificity, and a 0.76 AUC.

Radiomics has also been applied to study vertebral compression fractures (VCF), which can cause acute and chronic back pain. Wang et al achieved high AUC values (1.000 training, 0.964 test) for a CT-based radiomics model for VCF diagnosis.^57^ However, the perfect AUC in the training set may indicate overfitting. In patients with diabetes, Qiu et al achieved similarly high values.^58^ They analyzed opportunistic abdominal CT scans of 149 patients with diabetes and identified 12 features that were most predictive of abnormal bone mass through Lasso and Minimum Redundancy Maximum Relevance (MRMR) feature selection methods. The combined model with radiomic features with clinical features (eg, vertebral density) had an AUC of 0.95 in the validation cohort, while the radiomics-only model had an AUC of 0.90. The clinical significance of this work is that vertebral fractures are a common source of acute back pain in the diabetic patient population, with fractures often occurring with minimal trauma.^59^ The ability to identify risk enables preventative measures to be implemented earlier.

Similarly, Biamonte et al showed that vertebral fractures are significantly associated with radiomic features.^38^ After correcting for age, they found that vertebral fractures were significantly associated with radiomic features such as low-gray level zone emphasis (LGLZE), gray level non-uniformity (GLN) and neighboring gray-tone difference matrix (NGTDM) contrast. No significant differences in LGLZE (*P* = 0.94), GLN (*P* = 0.90), and NGTDM contrast (*P* = 0.54) were found between fractured subjects with osteoporotic BMD T-scores (≤−2.5 SD) and those with non-osteoporotic BMD T-scores, therefore suggesting that these radiomic features cannot distinguish between fracture patients with and without densitometric osteoporosis. Other studies have developed radiomic models to identify osteoporosis with varying AUC values (Table 4).^60-62^

### Prognostic and Treatment Response Modeling

Radiomics has been applied to predict vertebral fractures before they occur. Gui et al developed a model to treat predict radiation-induced VCF after spine stereotactic body radiation therapy (SBRT).^63^ They combined radiomic features from CT and T1 MRI with patient factors and found the random forest classification model could predict 1-year post-SBRT fractures with ∼84% sensitivity and 80% specificity. We have already discussed Qiu’s model predicting low bone mass in diabetics and Biamonte’s model identifying patients with fragility fractures.^58,64^ Thus, identifying risk via radiomics has downstream prognostic significance for pain and quality of life.

Radiomics has also been applied to guide treatment decision-making. Yu et al created a treatment prediction scheme for lumbar disc herniation using a radiomics-based nomogram that combined clinical features with imaging biomarkers.^65^ The combined radiomics-clinical nomogram constructed from 156 patients and 11 radiomic features had an AUC of 0.93 and an accuracy of 91%. They also calculated a “Rad-score”, where a higher “Rad-score” indicated imaging characteristics more inclined towards requiring surgical intervention. Saravi et al (2023) conducted a study on 172 patients undergoing microdiscectomy for lumbar disc herniation.^66^ They evaluated seventeen machine-learning pipelines and found that radial-basis-function neural network (RBF-NN) and a multilayer perceptron (MLP) yielded the strongest discrimination. Incorporating radiomics raised the RBF-NN AUC from 0.970 (clinical-only) to 0.992 and the MLP AUC from 0.785 to 0.832.

Radiomics has been used for predicting tumor and pain response after Spine Stereotactic Radiotherapy (SBRT), which is used to control tumor growth and alleviate pain. However, responses vary, and some patients develop post-SBRT VCFs or persistent pain. Chen et al built radiomics models using pre-treatment MRI of spinal metastases to predict treatment outcomes after SBRT.^67^ Here, outcomes were defined via the revised Response Evaluation Criteria in Solid Tumors (RECIST 1.1) criteria, where patients can be classified as progressive disease (PD) or non-PD after SBRT. They included 194 patients with spinal metastases who underwent SBRT and extracted 2264 radiomic features. The clinical model had an AUC of 0.733 while radiomics model had an AUC of 0.745-0.825 depending on whether T1-weighted, T2-weighted, or fat-suppressed T2-weighted sequences were used. The combined model with all clinical and radiomic features achieved the highest performance with an AUC of 0.828.

However, radiomic or clinical-radiomic combined models do not always exhibit better predictive performance than clinical models. Llorián-Salvador et al investigated predicting pain palliation response (responsive vs not responsive) after palliative radiation for spinal metastases.^68^ They extracted radiomic and semantic (eg, plastic reaction, posterolateral involvement of the spinal elements) features from CT scans, and found that the model based on established clinical parameters (eg, initial pain score, performance status) was the most predictive of pain relief with an AUC of ∼0.80. The radiomics and radiomics-clinical combined models had lower AUCs of 0.62 and 0.74 respectively. This suggests that imaging features from planning CT did not add much value in predicting pain response beyond clinically established parameters. Results here are similar to what was achieved by Saravi et al, who found minimal, but detectable, improvements in predictive tasks when radiomics features are included.^66^ This reinforces the existing opinion that each clinical use scenario may be impacted differently by the use of radiomics.

Radiomics has also been applied to predict outcomes in non-surgical treatments for back pain. Climent-Peris et al investigated MRI texture features as predictors of outcomes in non-specific chronic low back pain patients undergoing a standardized rehabilitation program.^69^ Radiomic features were extracted from lumbar discs, endplates, and paraspinal muscles on routine MRI scans, and a random forest model predicted which patients would fail to improve (≤30% pain reduction) after 6 months of rehabilitation. The radiomic model achieved a sensitivity of 86% and specificity of 57% (AUC ∼0.71), but interestingly, only had an AUC of ∼0.52 for patients with persistent disability. Similar work was done by Wakabayashi et al, who developed a radiomics-based model to predict pain response to radiation in 69 patients with painful spinal metastases.^70^ Their random forest model using combined clinical and radiomics data achieved an AUC of 0.848 for predicting significant pain relief (Table 5).

## Limitations in Current Studies

Although there is an increasing amount of evidence and interest in applying radiomics to a clinical context, there exist several key limitations that prevent this from happening at present. Few studies have directly examined how radiomic features themselves change over time in individuals with back pain. It has been noted that quantitative radiomic features enable objective longitudinal studies of low back pain, but this remains a theory.^56^ There have also been few studies investigating the association between radiomic markers and functional/performance metrics such as mobility and strength.

There is also a lack of demographic diversity in study populations. Most studies have been conducted in homogeneous populations with limited representation of different ethnicities and age groups. This leaves a gap in our understanding of the radiomic indicators that precede back pain, particularly in elderly populations where back pain prevalence is highest. Gender-based research disparities also exist, with several studies either not reporting sex-specific analyses or having insufficient representation of either sex.^52,65,66,71,72^ This is particularly problematic given existing evidence of sex-specific differences in paraspinal muscle morphology and function.^71,73,74^

So far, pain has been broadly categorized. Metrics used in studies do not capture fluctuations, chronicity nuances, or psychosocial dimensions (fear-avoidance, depression) that modulate pain outcomes. Radiomics alone cannot capture these, so any model purely based on imaging will have an upper limit of predictive capability for pain which is influenced by non-imaging factors.

Though many published models have performance metrics with AUCs greater than 0.9 in some cases, most lack external validation.^35,56^ Overfitting is a substantial concern due to the high dimensionality of radiomic data relative to sample size. Thus, the AUC in a real-world scenario may drop in independent cohorts. Until validated, they should be viewed as hypothesis-generating rather than definitive.

A general concern in the field of radiomics is the lack of standardization in image acquisition protocols, segmentation techniques, and feature extraction methodologies. While reproducibility reporting has improved, there remains significant heterogeneity in methodological approaches. This inconsistency makes direct comparison between studies difficult and hinders the development of generalizable prediction models.^35^

Technical limitations also exist. While deep learning approaches have improved the automated segmentation process, the variability in ROI definition across studies creates inconsistencies in feature extraction.^49^ Some researchers focus strictly on specific muscle boundaries, while others include fascial planes or do not excluding intramuscular fat. These methodological differences can substantially impact the extracted radiomics features and subsequent model performance across different datasets. Feature selection and dimensionality reduction techniques also vary considerably across studies. The frequent use of different machine learning algorithms with varying hyperparameter optimization strategies creates a fragmented methodological landscape. This inconsistency complicates the establishment of standardized clinical workflows and limits reproducibility.^35^

## Future Directions

For radiomics to impact patient care, tools must be integrated into existing radiology workflows. This means developing radiomics software that is compatible with hospital Picture Archiving and Communication Systems (PACS) and can process images in a way that is not resource intensive. Further, a physician-friendly interface for interpreting data should be developed, as radiomics workflows require interdisciplinary expertise that exceeds the training of most radiologists and clinicians. Radiomics algorithms will also need to be optimized for speed and reliability so that they can run on standard clinical hardware in real-time. Cloud-based solutions could also be explored.

To improve generalizability of radiomics models to different studies, hospitals, and imaging systems, future radiomics studies should be multicenter and international, with different scanners, protocols, and patient demographics present. During data analysis, we recommend stratified performance and calibration by sex, age, and race/ethnicity, and pre-specified subgroup analyses with material-difference thresholds and corrective strategies (eg, reweighting or group-aware calibration) when gaps are detected. If synthetic data augmentation is used, authors should detail the generator(s), validation, and bias/shift checks, recognizing emerging evidence that medical-imaging AI can inadvertently encode sensitive attributes (eg, race) and exhibit underdiagnosis in certain groups. To enable cross-study comparability, we advocate pre-specifying patient-centered outcomes with accepted thresholds for minimal clinically important difference (MCID) and consistent timepoints. For pain, use a 10-point numeric rating scale (NRS) with MCID ≈ 2-3 points or ≥30% improvement; for function, use the Oswestry Disability Index (ODI) version 2.1a with MCID ≈10 points or ≥30% improvement; for PROMIS domains (Pain Interference, Physical Function), target minimally important difference (MID) values of ∼2-3 T-score points.^75-80^ Models should, where feasible, incorporate psychosocial covariates (eg, STarT Back Tool for risk stratification) to better reflect the biopsychosocial nature of outcomes and to avoid overstating the standalone value of image-only predictors.^81,82^ Studies with stratified sampling across age groups, sexes, and ethnicities would improve generalizability and potentially identify population-specific radiomics signatures. External validation in diverse clinical settings should also become standard practice. Specifically, we recommend that clinical readiness be defined as achieving external validation across at least 3-5 independent centers and demonstrate AUC degradation of less than 0.05-0.10 from internal to external validation, and showing positive net benefit in decision curve analysis across clinically relevant threshold probabilities.^83-86^ The ability of radiomic models to reliably identify physiological markers across different scanners, institutions, and patient populations is critical for widespread clinical adoption. Establishing open-access datasets with standardized acquisition protocols would facilitate validation efforts and accelerate progress in the field. Longitudinal studies tracking radiomics features over time, particularly in high-risk populations such as those with occupation-specific exposures, would help establish the temporal relationship between tissue changes and symptom development. The integration of radiomics with other clinical data modalities, such as genomic data and electromyography, could improve predictive accuracy and provide a more comprehensive overview of back pain mechanisms. Studies should report minimal technical parameters including scanner manufacturer and model, field strength for MRI, reconstruction algorithms, sequence parameters (eg, repetition time, echo time), voxel dimensions, and any preprocessing steps applied.

Research should also move beyond diagnostic accuracy to investigate whether radiomic-guided interventions could produce meaningful clinical benefits. This includes randomized controlled trials comparing standard care to radiomic-informed treatment protocols, studies evaluating whether early risk stratification prevents complications, and research examining cost-effectiveness of implementing radiomics in routine clinical practice. To illustrate this necessity, despite nearly 8000 radiomics studies published in oncology since 2012, and some radiomics tools having received FDA clearance (such as QuantX for breast cancer diagnosis in 2017), few have achieved routine adoption in clinical practice.^87^ Further, while studies, such as those discussed above, have demonstrated that radiomics can identify diabetics at high risk for vertebral compression fractures, there is limited evidence showing whether early identification leads to effective preventive interventions or reduced fracture rates. Similarly, although radiomics can quantify paraspinal muscle fatty infiltration as a pain biomarker, studies have not yet demonstrated whether this knowledge changes treatment approaches beyond standard physical therapy prescriptions or improves patient outcomes. Identifying ways in which radiomics can be integrated into patient care routines will expedite its clinical adoption and increase its clinical utility.

## Conclusion

This review has explored the methodological framework of radiomics and its emerging applications in back pain assessment. Published works have demonstrated that radiomics can effectively characterize both soft tissue abnormalities such as paraspinal muscle degeneration and fascial changes, and hard tissue pathologies such as intervertebral disc degeneration and vertebral integrity. Furthermore, radiomics models have shown capability in predicting treatment outcomes across various interventions. Radiomics and combined clinical-radiomics models often, but not always, outperform models based on traditional clinical predictors.

Yet, challenges remain before radiomics can be widely implemented in clinical back pain management. Future technical work should focus on establishing standardized protocols for radiomics algorithms, developing user-friendly clinical interfaces, and creating models that are generalizable across different imaging modalities, conditions, and patient populations. Clinical research-wise, the methods in which radiomics can be appropriately integrated into clinical care should be investigated.

## Data Availability

Data sharing not applicable to this article as no datasets were generated or analyzed during the current study.
